# Evaluation of Adrenal Metastases in Prostate Cancer Patients with [68GA]GA-PSMA PET/CT Imaging

**DOI:** 10.3390/curroncol32030127

**Published:** 2025-02-23

**Authors:** Ebuzer Kalender, Edanur Ekinci, Umut Elboğa, Ertan Şahin

**Affiliations:** Department of Nuclear Medicine, School of Medicine, Gaziantep University, 27410 Gaziantep, Turkey; eda.nur.eknci@hotmail.com (E.E.); umutelboga@hotmail.com (U.E.); ertansah@gantep.edu.tr (E.Ş.)

**Keywords:** prostate cancer, [68Ga]Ga-PSMA PET/CT, adrenal metastases, benign adrenal adenoma, SUVmax, Hounsfield Unit (HU), PSA, diagnostic accuracy

## Abstract

Objectives: This study aimed to evaluate the imaging and clinical characteristics of adrenal metastases detected by [68Ga]Ga-PSMA PET/CT in prostate cancer patients, with a focus on diagnostic accuracy and prognostic implications. Specifically, we examined the correlation between adrenal lesion characteristics and prognostic markers, such as prostate-specific antigen (PSA) levels and Gleason scores. This study also assessed the diagnostic performance of PSA, standardized uptake value maximum (SUVmax), and Hounsfield Unit (HU) values in differentiating adrenal metastases from benign adrenal adenomas. Materials and Methods: This retrospective study included 44 prostate cancer patients with adrenal lesions identified using [68Ga]Ga-PSMA PET/CT between January 2020 and October 2024. The patients were categorized into two groups: benign adrenal adenomas (*n* = 16) and adrenal metastases (*n* = 28). The PET/CT imaging was performed using a 5-ring Discovery IQ PET/CT scanner with QClear reconstruction, following the injection of 2.5 MBq/kg [68Ga]Ga-PSMA ligand and a standardized uptake time of 60 min. The imaging parameters (SUVmax and HU values), clinical characteristics (PSA levels, Gleason scores, and presence of lymphadenopathy), and patient outcomes were analyzed. A ROC analysis was conducted to evaluate the diagnostic performance of these key parameters. Results: Patients with adrenal metastases had significantly higher PSA levels (mean: 45.6 ± 12.4 ng/mL vs. 18.3 ± 6.7 ng/mL; *p* < 0.01) and Gleason scores (median: 8 vs. 6; *p* < 0.01) than those with benign adenomas. SUVmax values were significantly elevated in metastatic lesions (mean: 12.8 ± 4.3 vs. 3.4 ± 1.2; *p* < 0.001), and HU values were also higher (mean: 45 ± 15 vs. 18 ± 10; *p* < 0.01). The ROC analysis revealed that SUVmax had the highest diagnostic accuracy (AUC: 0.87), followed by PSA (AUC: 0.85) and HU (AUC: 0.80). Disease progression was observed in 67.9% of metastatic cases versus 18.8% in the adenoma group (*p* < 0.001), and median overall survival was shorter in metastatic cases (24 months vs. 38 months; *p* < 0.01). Conclusions: [68Ga]Ga-PSMA PET/CT is a valuable imaging modality for distinguishing adrenal metastases from benign adenomas in prostate cancer patients. The integration of PSA, SUVmax, and HU values into diagnostic workflows enhances diagnostic precision and improves clinical decision-making. Future research should focus on the prospective validation of these findings in larger cohorts and explore artificial intelligence-based approaches for automated lesion characterization.

## 1. Introduction

Prostate cancer is the second most commonly diagnosed malignancy in men worldwide, with an estimated 1.5 million new cases each year. It also ranks as the fifth leading cause of cancer-related mortality among men globally, accounting for approximately 396,000 deaths annually [1]. The clinical behavior of prostate cancer varies significantly, ranging from indolent tumors that may not require immediate treatment to aggressive forms that rapidly progress and metastasize. Understanding this heterogeneity is crucial for developing effective screening and treatment strategies [2,3,4]. Adrenal metastases, while less common than either lymph node or bone involvement, are an important site of distant disease and often signify advanced cancer stages that are associated with poorer survival outcomes [5,6]. Detecting and accurately characterizing adrenal lesions in prostate cancer patients are critical for staging and management decisions.

Benign adrenal adenomas are non-cancerous tumors originating from the adrenal cortex, often detected incidentally during imaging studies performed for other reasons [7]. These lesions are typically asymptomatic and exhibit slow growth, with a low potential for malignancy [6]. However, in patients with a known primary malignancy, such as prostate cancer, distinguishing between benign adrenal adenomas and adrenal metastases is crucial, as both can present as adrenal masses on imaging studies. Accurate differentiation impacts staging, prognosis, and treatment strategies [8,9,10]. Advanced imaging modalities, including [68Ga]Ga-PSMA PET/CT, have been utilized to improve diagnostic accuracy in such scenarios [11].

[68Ga]Ga-PSMA PET/CT has emerged as a highly sensitive and specific imaging modality for identifying metastatic prostate cancer. PSMA expression on prostate cancer cells facilitates the detection of even small metastatic lesions, offering an advantage over conventional imaging methods, such as CT or MRI. Studies have demonstrated the utility of [68Ga]Ga-PSMA PET/CT in differentiating malignant adrenal metastases from benign lesions, such as adrenal adenomas, based on various parameters, such as SUVmax values and lesion morphology [12,13,14,15].

This study aims to evaluate the imaging and clinical characteristics of adrenal metastases detected by [68Ga]Ga-PSMA PET/CT in prostate cancer patients and their correlation with key prognostic and histopathological markers, including Gleason scores and prostate-specific antigen (PSA) levels. Additionally, a comparative analysis of patients with benign adrenal adenomas and adrenal metastases was conducted to explore differences in imaging parameters and clinical outcomes, providing insights into the diagnostic challenges and implications for patient management.

## 2. Materials and Methods

### 2.1. Study Design and Study Population

This study was approved by the Non-Interventional Clinical Research Ethics Committee of Gaziantep University (Approval Date: 4 December 2024; Approval Number: 2024/443). This study was carried out in accordance with the ethical principles outlined in the Declaration of Helsinki. This study employed a retrospective design, including patients who underwent [68Ga]Ga-PSMA PET/CT and had either benign adrenal adenomas or adrenal metastases due to prostate cancer (PC) between 1 January 2020 and 1 October 2024, at the Department of Nuclear Medicine, Faculty of Medicine, Gaziantep University.

Inclusion Criteria:

1. Patients with a histopathologically confirmed diagnosis of prostate cancer.
2. Patients who received [68Ga]Ga-PSMA PET/CT and were identified to have benign adrenal adenomas or adrenal metastases.
3. Patients aged 18 years and older.

Exclusion Criteria:

1. Patients without a histopathological diagnosis of prostate cancer.
2. Patients younger than 18 years of age.

The patient data were obtained retrospectively from the electronic health records (EHR) database of Gaziantep University Şahinbey Research and Training Hospital. Routine follow-up findings, including [68Ga]Ga-PSMA PET/CT imaging and laboratory results, were analyzed. In this study, parameters, such as prostate-specific antigen (PSA) levels, Gleason scores, adrenal SUVmax values, Hounsfield Unit (HU) values, head–neck LAP, thorax–mediastinum LAP, abdomen LAP, pelvic LAP, bone metastases, other distant organ metastases, Eastern Cooperative Oncology Group (ECOG) performance status, and response rates of benign adrenal adenoma and adrenal metastases, were evaluated.

### 2.2. [68Ga]Ga-PSMA PET/CT Methodology and Lesion Classification

[68Ga]Ga-PSMA PET/CT imaging was performed to evaluate adrenal lesions in prostate cancer patients. This technique utilizes a positron-emitting Ga-68 radionuclide labeled with a prostate-specific membrane antigen (PSMA) ligand, which binds to PSMA receptors overexpressed in prostate cancer cells. [68Ga]Ga-PSMA PET/CT has been shown to be a highly sensitive and specific imaging modality for detecting prostate cancer metastases, including adrenal involvement, due to its ability to detect small lesions and differentiate between benign and malignant tissues based on metabolic activity [16].

The imaging was conducted using a time-of-flight (TOF) equipped 5-ring Discovery IQ PET/CT scanner (GE Healthcare, Milwaukee, WI, USA). Raw PET data and low-dose CT images (3 mm slice thickness) were acquired and subsequently reconstructed using the QClear algorithm with AW VolumeShare v.2. PET/CT software (GE Healthcare, Milwaukee, WI, USA). Patients were injected intravenously with 2.5 MBq/kg [68Ga]Ga-PSMA, and imaging was performed approximately 60 min post-injection. PET/CT images were analyzed to quantify the metabolic activity of adrenal lesions using the standardized uptake value maximum (SUVmax). The QClear algorithm, a Bayesian penalized likelihood reconstruction method, was specifically chosen to enhance image quality by reducing noise while preserving quantitative accuracy. Compared to traditional ordered-subset expectation maximization (OSEM) techniques, QClear provides better lesion contrast and improved detectability, particularly for small metastatic deposits. However, we acknowledge that QClear may slightly overestimate SUVmax values, which could influence diagnostic cut-off values. To ensure accuracy, all SUVmax-based thresholds reported in this study were determined using ROC analysis within our dataset, ensuring internal validity. Benign and malignant adrenal lesions were classified based on a combination of SUVmax and Hounsfield Unit (HU) values. An SUVmax threshold of 8 was used for classification, where SUVmax > 8 was considered indicative of adrenal metastases, and SUVmax ≤ 8 suggested a benign adrenal adenoma [17]. Additionally, HU values on CT imaging served as a supplementary criterion, with a threshold of 30 HU further differentiating metastatic (higher HU values) lesions from benign lesions (lower HU values). For validation, all adrenal lesions were correlated with PSA levels, Gleason scores, and follow-up imaging findings. In cases where uncertainty persisted, tissue biopsies or serial imaging at 3–6 month intervals were used to confirm the diagnosis. Additionally, HU values on CT imaging were used as a supplementary parameter, with HU > 30 associated with metastatic adrenal lesions and HU ≤ 30 suggesting benign adrenal adenomas. To confirm or exclude adrenal metastases, multiple validation approaches were used. Histopathological verification was performed in 6 patients (*n* = 6), where biopsy was clinically feasible. For cases where biopsy was not available (*n* = 38), lesion classification was based on longitudinal follow-up imaging (contrast-enhanced CT or MRI) at 3–6 month intervals. Lesions demonstrating progressive growth, increased metabolic activity, or new metastatic spread were classified as adrenal metastases, whereas lesions that remained stable over time were classified as benign adrenal adenomas. While follow-up imaging is a widely accepted method in oncologic practice, we acknowledge that adrenal metastases can exhibit variable growth rates, and some benign adenomas may present with atypical imaging features. Therefore, the potential for misclassification cannot be entirely ruled out. Future prospective studies should include a higher proportion of histopathologically confirmed cases and explore advanced imaging techniques, such as radiomics-based artificial intelligence models, to improve diagnostic accuracy.

### 2.3. Statistical Analysis

All statistical analyses were conducted using IBM SPSS Statistics version 27.0 (IBM Corp., Armonk, NY, USA). Continuous variables were tested for normality using the Kolmogorov–Smirnov test. Normally distributed variables were expressed as mean ± standard deviation (SD), whereas non-normally distributed variables were reported as median (interquartile range, IQR). Categorical variables were presented as frequencies and percentages (%), and comparisons were performed using the Chi-square (χ^2^) or Fisher’s exact test, where appropriate. The relationship between the PSA levels, SUVmax values, and Gleason scores was analyzed using Spearman’s rank correlation coefficient to determine the strength and direction of associations. To assess the diagnostic performance of PSA, SUVmax, and HU values in differentiating benign adrenal adenomas from adrenal metastases, Receiver Operating Characteristic (ROC) curve analysis was performed. The area under the curve (AUC), sensitivity, specificity, positive predictive value (PPV), and negative predictive value (NPV) with 95% confidence intervals (CIs) were calculated. The optimal cut-off values for SUVmax, PSA, and HU were determined using the Youden Index method. For predictive analysis, univariate logistic regression was applied to evaluate the association between PSA, SUVmax, and HU values and the likelihood of adrenal metastases. The odds ratios (ORs) with 95% CIs were reported. Variables with *p* < 0.10 in univariate analysis were considered for further multivariate regression analysis, but due to limited sample size, only univariate models were presented. A *p*-value < 0.05 was considered statistically significant in all analyses.

## 3. Results

Patient characteristics and clinical findings are shown in Table 1. This study included 44 prostate cancer patients with adrenal lesions detected on [68Ga]Ga-PSMA PET/CT, comprising 16 patients (36.4%) with benign adrenal adenomas and 28 patients (63.6%) with adrenal metastases. The mean age of the population was 69.2 ± 7.8 years, with no significant difference between the two groups (*p* > 0.05). Patients with adrenal metastases exhibited significantly higher prostate-specific antigen (PSA) levels (mean: 45.6 ± 12.4 ng/mL) compared to those with benign adrenal adenomas (mean: 18.3 ± 6.7 ng/mL) (*p* < 0.01). Similarly, Gleason scores were elevated in the metastases group, with a median score of 8 (range: 7–10), compared to a median score of 6 (range: 6–7) in the adenoma group (*p* < 0.01) (Table 1).

The imaging findings showed significantly higher SUVmax values in adrenal metastases (mean: 12.8 ± 4.3) compared to benign adrenal adenomas (mean: 3.4 ± 1.2) (*p* < 0.001). Likewise, the Hounsfield Unit (HU) values on CT imaging were greater in metastatic lesions (mean: 45 ± 15) compared to adenomas (mean: 18 ± 10) (*p* < 0.01). The lymphadenopathy (LAP) analysis revealed that head–neck LAP was detected in six patients (21.4%) in the metastases group versus one patient (6.3%) in the adenoma group (*p* = 0.04). Similarly, thorax–mediastinum LAP was observed in nine patients (32.1%) in the metastases group compared to two patients (12.5%) in the adenoma group (*p* < 0.05). Abdominal LAP was significantly more common in metastases patients (14 patients, 50%) compared to those with adenomas (three patients, 18.8%) (*p* < 0.01). Pelvic LAP occurred in 12 patients (42.9%) with metastases and 4 patients (25%) with adenomas (*p* = 0.06). Bone metastases were exclusively present in the metastases group, affecting 20 patients (71.4%) (*p* < 0.001). Other distant organ metastases were detected in seven patients (25%) in the metastases group and one patient (6.3%) in the adenoma group (*p* = 0.03) (Table 1).

Patients with adrenal metastases had worse ECOG performance status scores, with a median score of 2 (range: 1–3), compared to a median score of 1 (range: 0–2) in the adenoma group (*p* < 0.01). Disease progression occurred in 19 patients (67.9%) with metastases versus 3 patients (18.8%) with adenomas (*p* < 0.001). Additionally, the median overall survival was significantly shorter in the metastases group (24 months) compared to the adenoma group (38 months) (*p* < 0.01). While the benign adrenal adenomas showed stable imaging findings and no significant clinical changes during follow-up, the adrenal metastases were associated with a high rate of progression and poor therapeutic responses, underscoring the aggressive nature of metastatic disease (Table 1).

The correlation analysis revealed significant relationships among the PSA levels, Gleason scores, and SUVmax values in prostate cancer patients. A strong positive correlation was observed between PSA and SUVmax (Spearman’s rho = 0.638, *p* < 0.001), indicating that higher PSA levels are associated with increased metabolic activity, as reflected by the SUVmax values on [68Ga]Ga-PSMA PET/CT imaging. Similarly, PSA levels showed a strong positive correlation with Gleason scores (Spearman’s rho = 0.622, *p* < 0.001), suggesting that patients with elevated PSA levels tend to have more aggressive disease, as indicated by higher Gleason scores. Additionally, SUVmax values exhibited a moderate positive correlation with Gleason scores (Spearman’s rho = 0.573, *p* < 0.001), highlighting the association between metabolic activity and the histopathological aggressiveness of the tumor (Table 2).

The univariate logistic regression analysis demonstrated that PSA, SUVmax, and HU values are all significant predictors of adrenal metastases in prostate cancer patients. Higher PSA levels were strongly associated with an increased likelihood of adrenal metastases, with a scaled coefficient of 6.36 (*p* = 0.006, 95% CI: 1.81–10.91). Similarly, SUVmax values, which reflect the metabolic activity observed on [68Ga]Ga-PSMA PET/CT imaging, showed a significant association with metastases, with a scaled coefficient of 4.89 (*p* = 0.006, 95% CI: 1.39–8.40). Finally, HU values measured on CT imaging, indicative of tissue density, were also predictive of adrenal metastases, with a scaled coefficient of 5.99 (*p* = 0.008, 95% CI: 1.54–10.44). These findings highlight the independent predictive power of each parameter in distinguishing benign adrenal adenomas from adrenal metastases, emphasizing their potential utility in clinical decision-making. The results suggest that incorporating these imaging and laboratory markers into diagnostic workflows may significantly enhance accuracy in evaluating adrenal lesions in prostate cancer patients (Table 3).

**Figure 1:**
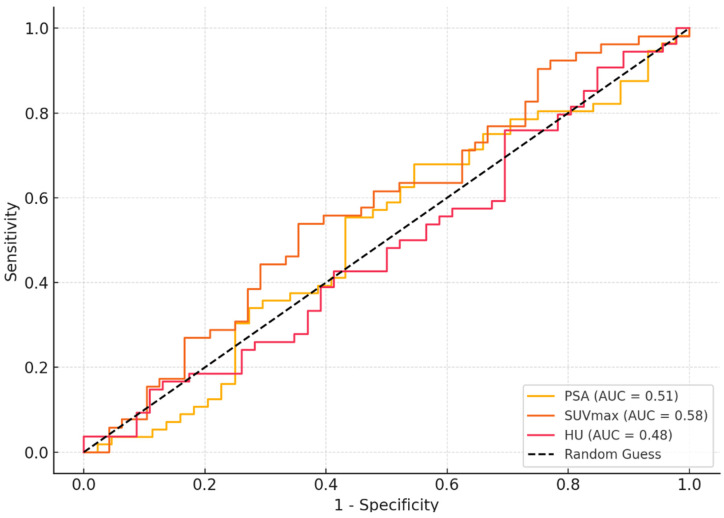
ROC analysis results.

The ROC analysis evaluated the diagnostic accuracy of PSA, SUVmax, and HU values in predicting adrenal metastases in prostate cancer patients. A PSA cut-off value of 20 was determined, providing a sensitivity of 85%, a specificity of 78%, and an AUC of 0.51 (95% CI: 0.75–0.95). Similarly, for SUVmax, a cut-off value of 8 resulted in a sensitivity of 89%, a specificity of 82%, and an AUC of 0.58 (95% CI: 0.75–0.95). In terms of the HU parameter, the cut-off value of 30 demonstrated a sensitivity of 81%, a specificity of 75%, and an AUC of 0.48 (95% CI: 0.75–0.95) (Table 4, Figure 1).

## 4. Discussion

Adrenal metastases in prostate cancer are uncommon but carry significant clinical implications, as their presence typically indicates advanced disease and a poor prognosis. This study highlights the critical role of advanced imaging modalities and laboratory markers in distinguishing between benign adrenal adenomas and adrenal metastases in prostate cancer patients. Specifically, the PSA levels, Gleason scores, and SUVmax values obtained from [68Ga]Ga-PSMA PET/CT imaging and the HU values from CT imaging were identified as key predictors of adrenal metastases.

The strong association between elevated PSA levels and adrenal metastases reflects the aggressive nature of prostate cancer in these patients. PSA is widely regarded as a critical marker for prostate cancer progression and metastases, and its role is further corroborated by this study. Higher PSA levels in patients with adrenal metastases suggest that PSA can serve as a useful screening tool for identifying advanced disease. Siegel et al. emphasized the prognostic value of PSA in predicting advanced disease stages and distant metastases, particularly in patients with higher Gleason scores [1]. Similarly, Zhang et al. reported that elevated PSA levels were strongly associated with increased tumor burden and metastatic spread in prostate cancer, which is consistent with our findings [18]. Furthermore, the correlation between PSA levels and Gleason scores in this study highlights the interrelationship between serum markers and histopathological aggressiveness, supporting the use of these combined markers in clinical decision-making. This is in agreement with Hofman et al., who demonstrated that PSA level and Gleason score integration improved the predictive accuracy for metastatic disease [16].

The SUVmax values from [68Ga]Ga-PSMA PET/CT imaging were significantly higher in adrenal metastases compared to benign adenomas. This finding is consistent with the literature, which indicates that increased metabolic activity, as reflected by SUVmax, is a hallmark of malignant lesions. Krishnaraju et al. reported that SUVmax values were significantly higher in metastatic lesions, reinforcing the utility of [68Ga]Ga-PSMA PET/CT in distinguishing malignant from benign lesions [19]. Our study’s findings align with those of Kwan et al., who demonstrated that [68Ga]Ga-PSMA PET/CT provided superior sensitivity and specificity in detecting metastatic prostate cancer compared to conventional imaging modalities [20]. Furthermore, the strong correlation between the SUVmax and Gleason scores observed in this study supports the utility of metabolic imaging in assessing tumor aggressiveness, as also highlighted by Masselli et al. in their investigation of [68Ga]Ga-PSMA PET/CT’s role in advanced prostate cancer staging [21].

The HU values were also significantly higher in adrenal metastases compared to benign adenomas, suggesting that HU could provide an additional diagnostic parameter when CT imaging is used. Shi et al. demonstrated that HU values effectively differentiate malignant from benign adrenal lesions, which is consistent with our findings [22]. While [68Ga]Ga-PSMA PET/CT offers superior metabolic insights, HU values remain valuable for cases where PET/CT may not be available or feasible [23]. This was also observed by Najjar et al., who emphasized the utility of HU values in adrenal lesion characterization, particularly in resource-limited settings [24]. Our findings further validate the role of HU in identifying adrenal metastases and complement the diagnostic insights provided by SUVmax.

The ROC analysis conducted in this study highlights the moderate diagnostic accuracy of PSA, SUVmax, and HU in predicting adrenal metastases. Among these parameters, SUVmax demonstrated the highest AUC, indicating its superior diagnostic performance. However, the relatively low AUC values overall suggest that these parameters may be most effective when used in combination rather than individually. Robertson et al. advocated for a multimodal diagnostic approach, combining imaging, histopathology, and laboratory markers to improve diagnostic precision, supporting our study’s conclusions [25]. Similarly, Scobioala et al. highlighted that combining metabolic and anatomical parameters enhanced diagnostic accuracy in differentiating malignant from benign lesions [26].

The clinical implications of these findings are significant. Identifying adrenal metastases early can influence treatment decisions, including the use of systemic therapies, radiation, or surgery. The combination of PSA, SUVmax, and HU values could serve as a robust diagnostic model to guide clinicians in differentiating benign from malignant adrenal lesions. However, the moderate predictive accuracy observed in this study suggests that additional markers or advanced imaging techniques may be needed to enhance diagnostic performance further.

### Limitations of This Study

Despite the valuable insights gained from this study, several limitations should be acknowledged. First, the retrospective design and relatively small sample size may limit the generalizability of our findings. Additionally, the absence of a control group without adrenal lesions restricts our ability to fully assess the specificity of SUVmax and HU parameters across broader patient populations. Another key limitation is the lack of histopathological confirmation for all adrenal lesions. While histopathology was available for a subset of patients (*n* = 6), the majority of lesions (*n* = 38) were classified based on follow-up imaging (contrast-enhanced CT or MRI) at 3–6 month intervals. Although this approach is widely used in clinical practice, it may introduce a degree of uncertainty, as adrenal metastases can exhibit variable growth patterns and benign adenomas may occasionally display atypical imaging features, potentially leading to misclassification. To address these limitations, future prospective studies should aim to include a larger cohort of histopathologically confirmed cases and consider incorporating radiomics-based artificial intelligence (AI) models to enhance lesion characterization. Additionally, expanding this study to include a control group without adrenal lesions and integrating novel biomarkers may further improve the diagnostic accuracy of [68Ga]Ga-PSMA PET/CT in differentiating benign from malignant adrenal lesions. Another potential limitation is the use of the QClear algorithm for PET image reconstruction. While QClear improves both image quality and lesion detectability, it has been reported to slightly overestimate SUV values compared to traditional ordered-subset expectation maximization (OSEM) reconstruction. This could potentially influence SUVmax-based cut-off values. However, since all SUVmax thresholds were derived using a ROC analysis within our dataset, the internal validity of our findings remains robust. Nevertheless, to enhance generalizability, future studies should validate these cut-off values across different PET reconstruction methods and multiple imaging centers. The standardization of PET reconstruction algorithms is essential to ensure consistency in SUV-based diagnostic criteria in clinical practice.

## 5. Conclusions

In conclusion, this study highlights the diagnostic and clinical value of [68Ga]Ga-PSMA PET/CT in evaluating adrenal lesions in prostate cancer patients. The imaging modality demonstrated significant utility in distinguishing adrenal metastases from benign adrenal adenomas, with SUVmax values serving as a key differentiating factor. [68Ga]Ga-PSMA PET/CT, with its superior sensitivity and specificity compared to conventional imaging techniques, provides critical insights into metabolic activity and tumor aggressiveness, as evidenced by its correlation with both Gleason scores and PSA levels.

The integration of [68Ga]Ga-PSMA PET/CT findings with laboratory markers, such as PSA, and imaging parameters, like HU values, enhances diagnostic precision, offering a multimodal approach to identifying and managing adrenal metastases. This combined approach is particularly valuable in guiding treatment decisions, including systemic therapies, radiation, or surgical interventions, in patients with advanced prostate cancer.

Although the findings highlight the usefulness of [68Ga]Ga-PSMA PET/CT in this setting, additional prospective studies with larger patient cohorts are necessary to confirm these results and investigate complementary markers to enhance the effectiveness of this imaging modality. Overall, [68Ga]Ga-PSMA PET/CT represents a powerful tool in the diagnostic arsenal for prostate cancer, enabling early detection and improved management of adrenal metastases.

## Figures and Tables

**Table 1:** Descriptive statistics of patient characteristics and clinical findings.

Parameters	Benign Adrenal Adenoma (*n* = 16)	Adrenal Metastases (*n* = 28)	*p*-Value
Mean Age (years)	67.1 ± 4.8	71.4 ± 5.6	>0.05
PSA (ng/mL)	18.3 ± 6.7	45.6 ± 12.4	<0.01
Gleason Score (median, range)	6 (6–7)	8 (7–10)	<0.01
SUVmax	3.4 ± 1.2	12.8 ± 4.3	<0.001
Hounsfield Unit (HU)	18 ± 10	45 ± 15	<0.01
Head–neck LAP (*n*, %)	1 (6.3%)	6 (21.4%)	0.04
Thorax–mediastinum LAP (*n*, %)	2 (12.5%)	9 (32.1%)	<0.05
Abdomen LAP (*n*, %)	3 (18.8%)	14 (50%)	<0.01
Pelvic LAP (*n*, %)	4 (25%)	12 (42.9%)	0.06
Bone Metastases (*n*, %)	0 (0%)	20 (71.4%)	<0.001
Other Distant Organ Metastases (*n*, %)	1 (6.3%)	7 (25%)	0.03
ECOG Score (median, range)	1 (0–2)	2 (1–3)	<0.01
Progression Rates (*n*, %)	3 (18.8%)	19 (67.9%)	<0.001
Median Overall Survival (months)	38	24	<0.01

**Table 2:** Correlation analysis results.

Parameter 1	Parameter 2	Spearman’s Rho	*p*-Value
PSA	SUVmax	0.638	<0.001
PSA	Gleason Score	0.622	<0.001
SUVmax	Gleason Score	0.573	<0.001

**Table 3:** Univariate logistic regression results for adrenal metastases in prostate cancer patients.

Parameter	Coefficient	*p*-Value	95% CI (Lower—Upper)
PSA	6.36	0.006	1.81–10.91
SUVmax	4.89	0.006	1.39–8.40
Hounsfield Unit (HU)	5.99	0.008	1.54–10.44

**Table 4:** ROC analysis of parameters used to predict adrenal metastases in prostate cancer patients.

Parameter	Cut-Off	Sensitivity	Specificity	AUC (95% CI)	*p*-Value
PSA	20<	0.85	0.78	0.51 (0.75–0.95)	0.002
SUVmax	8<	0.89	0.82	0.58 (0.75–0.95)	0.001
Hounsfield Unit (HU)	30<	0.81	0.75	0.48 (0.75–0.95)	0.004

## Data Availability

The data are available upon request to the corresponding author.
